# Methodology of aiQSAR: a group-specific approach to QSAR modelling

**DOI:** 10.1186/s13321-019-0350-y

**Published:** 2019-04-03

**Authors:** Kristijan Vukovic, Domenico Gadaleta, Emilio Benfenati

**Affiliations:** 1 Istituto di Ricerche Farmacologiche Mario Negri-IRCCS, Via Mario Negri 2, 20156 Milan, Italy; 2 Jozef Stefan International Postgraduate School, Jamova cesta 39, 1000 Ljubljana, Slovenia

**Keywords:** QSAR, Local models, Machine learning, Applicability domain measure, BCF, Ames test, Acute oral toxicity

## Abstract

**Background:**

Several QSAR methodology developments have shown promise in recent years. These include the consensus approach to generate the final prediction of a model, utilizing new, advanced machine learning algorithms and streamlining, standardization and automation of various QSAR steps. One approach that seems under-explored is at-the-runtime generation of local models specific to individual compounds. This approach was quite likely limited by the computational requirements, but with current increases in processing power and the widespread availability of cluster-computing infrastructure, this limitation is no longer that severe.

**Results:**

We propose a new QSAR methodology: aiQSAR, whose aim is to generate endpoint predictions directly from the input dataset by building an array of local models generated at-the-runtime and specific for each compound in the dataset. The local group of each compound is selected on the basis of fingerprint similarities and the final prediction is calculated by integrating the results of a number of autonomous mathematical models. The method is applicable to regression, binary classification and multi-class classification and was tested on one dataset for each endpoint type: bioconcentration factor (BCF) for regression, Ames test for binary classification and Environmental Protection Agency (EPA) acute rat oral toxicity ranking for multi-class classification. As part of this method, the applicability domain of each prediction is assessed through the applicability domain measure, calculated on the basis of the fingerprint similarities in each local group of compounds.

**Conclusions:**

We outline the methodology for a new QSAR-based predictive tool whose advantages are automation, group-specific approach to modelling and simplicity of execution. Our aim now will be to develop this method into a stand-alone software tool. We hope that eventual adoption of our tool would make QSAR modelling more accessible and transparent. Our methodology could be used as an initial modelling step, to predict new compounds by simply loading the training dataset as an input. Predictions could then be further evaluated and refined either by other tools or through optimization of aiQSAR parameters.

**Electronic supplementary material:**

The online version of this article (10.1186/s13321-019-0350-y) contains supplementary material, which is available to authorized users.

## Introduction

The use of quantitative structure–activity relationship (QSAR) methods has expanded significantly in recent decades [1, 2]. Consequently—and due to the success of early QSAR models—there is growing interest in these methods from the regulatory perspective, which in turn influences further QSAR development [3, 4]. Some of the driving factors are certainly the very high costs—financially, but also timewise [5]—required for standard in vivo and in vitro regulatory tests, as well as ethical concerns related to the use of animals for in vivo tests [6].

Recent developments of QSAR methods show several characteristics. The first is the move towards an integrated approach in modelling [7]. This means that the final output of a model is a consensus of several predictions, each obtained by a distinct QSAR approach [8–10]. This is further evidenced by the structure of community-wide modelling efforts, such as the ongoing initiative organized by the acute toxicity workgroup (ATWG) of the Interagency Coordinating Committee on the Validation of Alternative Methods (ICCVAM) [11], which shifted the paradigm from the commonly used approach of selecting a best method, to an integrated approach, combining predictions (with different weights) of all models developed within the initiative.

The second characteristic is streamlining and automation of the workflow. The same shift can be observed in various fields that are emerging from big-data science, such as bioinformatics [12]. Briefly, automation and standardization tools are increasing in number, aiming to streamline some of the most frequently used steps in QSAR development. These range from general tools for model development [13, 14], to procedures that automatically manipulate datasets [15] and consequently, tools to simplify data conversions and interactions between various platforms used in different steps [16]. A very good outcome of these streamlining efforts is more transparency in QSAR model development, especially in terms of training set preparation [17] and methods for performance validation [18].

The last characteristic is the shift from simple, linear QSAR methods towards more sophisticated machine-learning algorithms, which were shown to perform significantly better [19, 20]. However, this comes at a cost. Linear regression provided a very straightforward way of interpreting the model since it was immediately obvious which descriptors contributed to prediction the most. For the majority of machine learning algorithms, getting the mechanistic interpretation is very hard—if not impossible. A similar issue is seen in many computer science fields, where the analogous “black box” behavior problem of these algorithms makes any backtracking of the mathematical procedure (and therefore the interpretation of results) unfeasible [21].

These are all established though under-explored methodologies [22]. Some examples are the Food and Drug Administration (FDA) method of the T.E.S.T. software [23] and the lazar framework [24, 25]. Our aim is to incorporate these developments into a new QSAR-based tool: aiQSAR. The tool should be automated so that it takes as an input the dataset of training compounds, regardless of the endpoint, and predicts values for new, unknown compounds. As a mathematical procedure, we employ an array of machine learning algorithms and integrate their predictions into the final output. The focus of the tool is on building distinct, local-group based models, specific to each compound in the dataset and generated at-the-runtime. Therefore, our models are built “from scratch” for each compound in the dataset, used for a single prediction, and the procedure is repeated from the beginning for the next compound.

## Methods

**Fig. 1:**
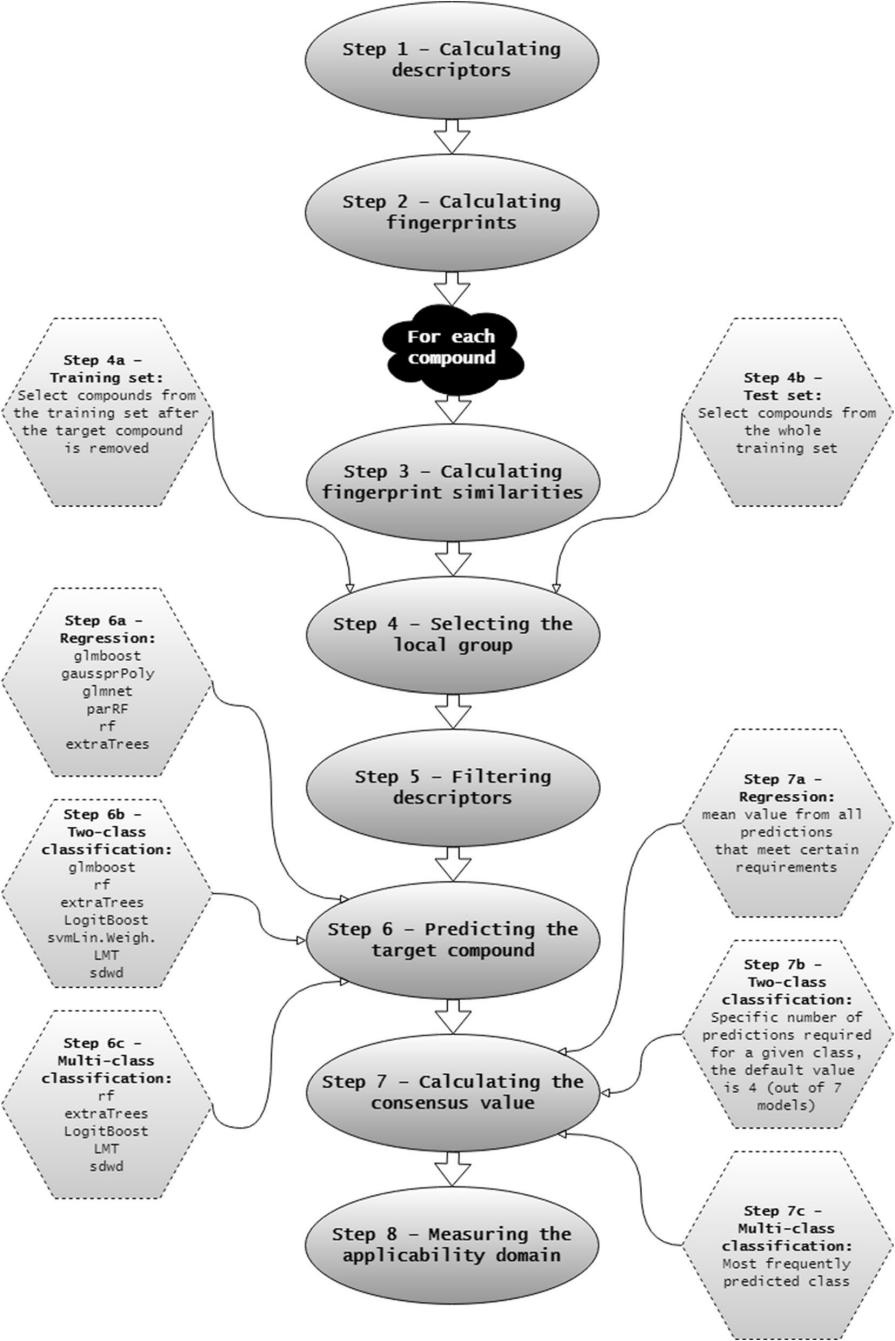
aiQSAR workflow

In this section we use the term “training set” (TS) to indicate the dataset of compounds used for model derivation whose endpoint values are known and the term “evaluation set” (ES) to indicate the list of compounds of the same endpoint whose values are unknown. Two distinct modes in which aiQSAR method can be employed are cross-validating (re-testing) the training set and predicting the endpoint values for compounds in the evaluation set. The workflow (Fig. 1, Additional file 1) can be divided into 8 steps:

*Step 1*–*Calculating descriptors* 3839 descriptors from Dragon 7 software [26] are calculated for all TS and ES compounds. All 1D and 2D available descriptors are selected. An alternative source of descriptors can be used and involves importing a numerical matrix of descriptors (calculated for all TS and ES compounds).

*Step 2*–*Calculating fingerprints* Fingerprints are calculated for all TS and ES compounds by the R package “rcdk” [27]. Two types of fingerprints, “pubchem” and “extended”, are obtained by the “get.fingerprint” function [28]. Pubchem fingerprints are structural keys defined by the Pubchem database [29]. Extended fingerprints are obtained through a published fingerprinting procedure [30] in which paths up to six chemical bonds of depth are considered and the resulting sequence is folded to the length of 1024 bits.

The following steps are carried out for each compound (referred to as “the target compound”) individually, for the training and the evaluation set:

*Step 3*–*Calculating fingerprint similarities* Tanimoto distance [31] is used as a measure of fingerprint similarities. The distance between the target compound and all TS compounds is calculated by the R package “fingerprint” [32].

**Fig. 2:**
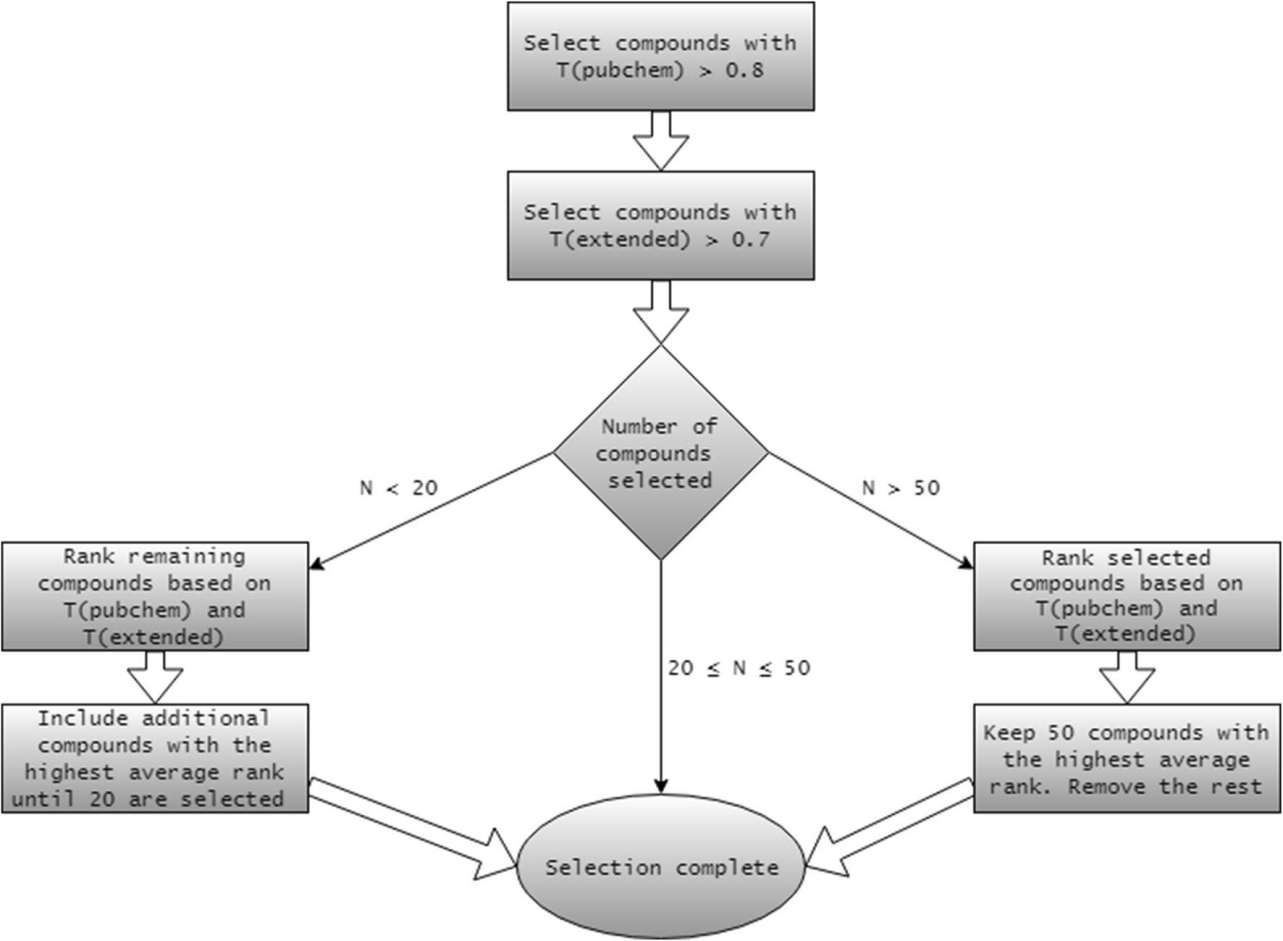
Selecting the local group of compounds. $T\left( {pubchem} \right)$ and $T\left( {extended} \right)$ are Tanimoto distances based on “pubchem” and “extended” fingerprints, respectively (see Step 2)

*Step 4*–*Selecting the local group* An algorithm (Fig. 2) is used to select between 20 and 50 TS compounds that are most similar to the target compound. This similarity is based on Tanimoto distances between fingerprints of the target compound and all TS compounds (Step 3): First, all compounds that meet initial criteria are selected. Then, if the number of compounds is not met, a further refinement is done based on the average ranks of two similarity measures.

*Step 4a*–*Training set* For a target compound in the TS, the local group of similar compounds is selected from the remainder of the TS after the target compound is removed.

*Step 4b*–*Evaluation set* For a target compound in the ES, the local group of similar compounds is selected from the whole TS.

*Step 5*–*Filtering descriptors* All descriptors with a missing value for any compound in the local group or for the target compound are discarded. Then zero-variance and near-zero-variance descriptors are removed using the “nearZeroVar” function from the R package “caret” [33]. It should be emphasized that this procedure is carried out independently for each target compound, so different descriptors are likely to be selected each time.

*Step 6*–*Predicting the target compound* Several mathematical models are built from the local group and each model is used to predict the endpoint value of the target compound. In this step, the R package “caret” is used. Different methods are used depending on the endpoint type. Specific methods were selected based on their performance in an independent evaluation of a large collection of datasets (data not shown). The names given here correspond to the respective methods in the “caret” training function [34].

*Step 6a*–*Regression* For regression endpoints, the following methods are used: glmboost (Boosted Generalized Linear Model) [35], gaussprPoly (Gaussian Process with Polynomial Kernel) [36], glmnet (Lasso and Elastic-Net Regularized Generalized Linear Models) [37], rf (Random Forest) [38], extratrees (Random Forest by Randomization) [39].

*Step 6b*–*Binary classification* For binary endpoints, the following methods are used: glmboost, rf, extraTrees, LogitBoost (Boosted Logistic Regression) [40], svmLinearWeights (Linear Support Vector Machines with Class Weights) [41], LMT (Logistic Model Trees) [42], sdwd (Sparse Distance Weighted Discrimination) [43].

*Step 6c*–*Multi*-*class classification* For multi-class endpoints, the following methods are used: rf, extraTrees, LogitBoost, LMT, sdwd.

*Step 7*–*Calculating the consensus value* Predictions from all methods are combined into a single output value. Different procedures are used depending on the endpoint type.

*Step 7a*–*Regression* First, we discarded predictions from any of the five individual models that are more than 10% higher than the overall highest TS value, or more than 10% lower than the lowest TS value. The final prediction is calculated as a mean of the remaining values predicted by individual models.

*Step 7b*–*Binary classification* The final prediction is the majority vote out of the seven individual models.

*Step 7c*–*Multi*-*class classification* The final prediction is the most frequently predicted class out of the five individual models. In case of a tie, the class that is least represented in the TS (out of those that are tied) is selected.

**Fig. 3:**
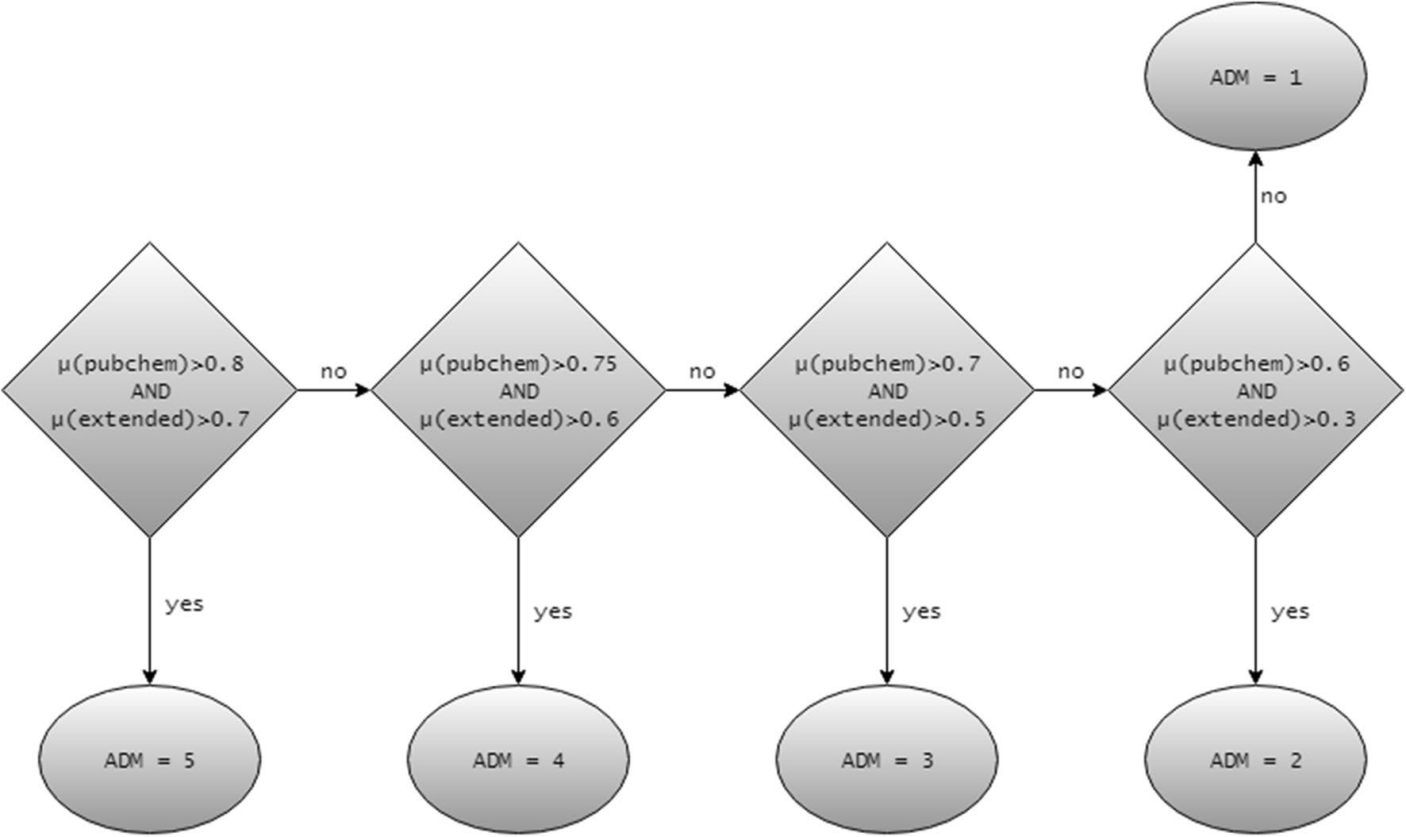
Calculating the ADM. $\mu \left( {pubchem} \right)$ and $\mu \left( {extended} \right)$ are average values of the Tanimoto distances between the target compound and compounds in its local group

*Step 8*–*Assessing the applicability domain* Applicability domain measure (ADM) for the target compound is computed based on similarities between the target compound and compounds in its local group (Fig. 3). The target compound is assigned a rank between 1 and 5. The higher average similarity with compounds from the local group corresponds to the higher ADM rank. This measure can therefore be used to assess the coverage of chemical space relevant to the class of the target compound and, on the basis of the desired ADM cutoff, either to accept the final prediction as reliable or to reject it.

## Results and discussion

The workflow was tested on a number of datasets. Three examples will be discussed, one for each endpoint type (regression, binary classification and multi-class classification).

### Regression

**Fig. 4:**
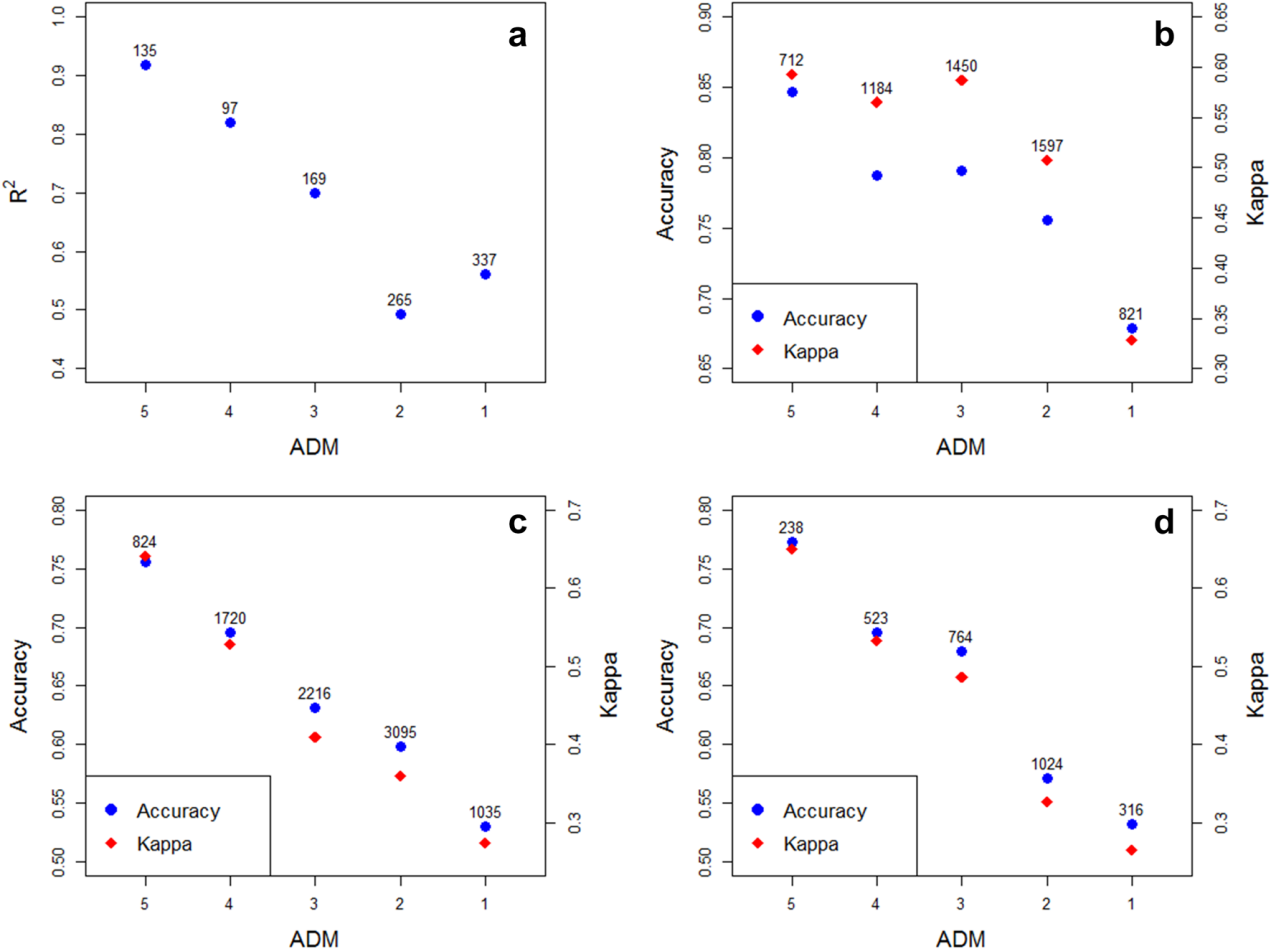
Plot of performance measures against the ADM. The figures above each point indicate the number of compounds in the corresponding ADM class. **a** Bioconcentration factor dataset, **b** Ames mutagenicity dataset, **c** EPA acute oral toxicity training set, **d** EPA acute oral toxicity evaluation set

The bioconcentration factor (BCF) was the endpoint tested for regression. The dataset was obtained from the VEGA software [44] (Additional file 2). BCF is the ratio of the concentration of a chemical in an organism to the concentration of that chemical in the surrounding environment in steady-state conditions [45]. Information on the dataset and performance statistics are given below (Table 1). In general, a higher ADM corresponds to greater average similarity between the target compound and compounds in its local group. Therefore, we would expect the method to perform better on compounds with a higher ADM. If we plot the R^2^ statistic against the ADM class (Fig. 4a), we get the expected positive correlation.

**Table 1:** Bioconcentration factor-dataset statistics and performance measures. RMSE stands for Root-mean-square error; MAE stands for Mean absolute error

Endpoint	BCF
Endpoint type	Regression
Number of compounds	1003
Range of values	− 1.70 to 6.06
RMSE	0.74
R^2^	0.72
MAE	0.53

### Binary classification

**Fig. 5:**
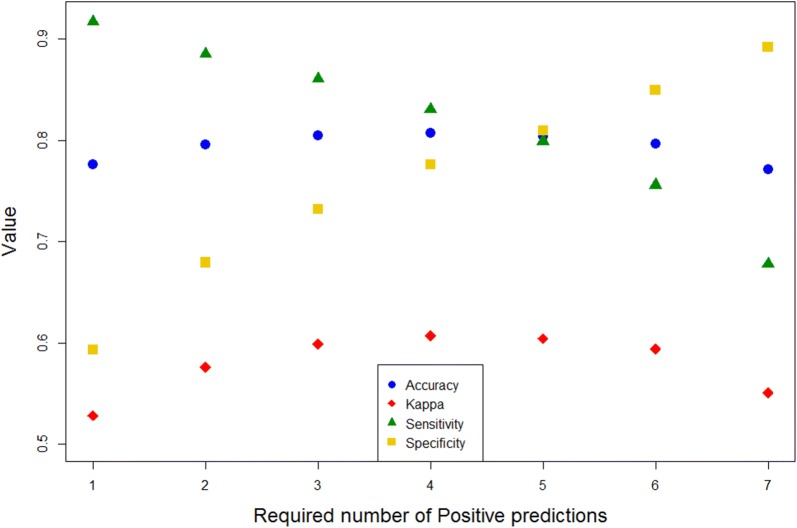
Various performance measures plotted against the required number of positive predictions for the Ames mutagenicity test dataset

Ames mutagenicity was the endpoint tested for binary classification. The dataset was obtained from the VEGA software [44] (Additional file 3). Ames test is a biological assay to assess the mutagenic potential of chemical compounds[46]. Information on the dataset and performance statistics are given below (Table 2). Again, there is a positive correlation between performance measures (Accuracy, Cohen’s Kappa –$\kappa$–) and ADM (Fig. 4b). Since the modelling procedure for binary classifications consists of building up seven different models, the default requirement for consensus is four predictions of the same class (Fig. 1). However, we can assess the performance of the method in relation to this requirement by varying the number of predictions required for a given class (in this case—Positives; Fig. 5). In this example our method performs best with the default cutoff of 4. More broadly, this way we can artificially raise or lower the likelihood of certain statistical errors (*e.g.* false positives, false negatives). We might want to do this if our model has some specific application. For example, if it is used in a regulatory context it would be reasonable to reduce the default requirement on positives in order to minimize the number of false negatives. We also noticed that for some highly unbalanced datasets reducing this requirement in favor of the minority class actually improved the overall performance.

**Table 2:**
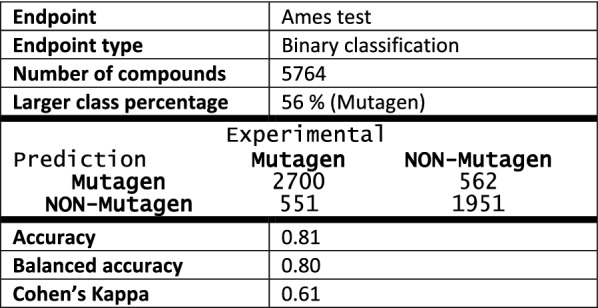
Ames mutagenicity test—dataset statistics, confusion matrix and performance measures

### Multi-class classification

Environmental Protection Agency (EPA) ranking for the acute oral toxicity in rats was the endpoint tested for multi-class classification. This dataset was analyzed as part of the acute oral systemic toxicity modelling initiative launched by the NTP Interagency Center for the Evaluation of Alternative Toxicological Methods (NICEATM) [47]. The objective of this initiative was predictive modelling of five endpoints related to acute toxicity and measured as rat oral LD_50_ [48]: very toxic (binary classification), nontoxic (binary classification), LD_50_ point estimates (regression), EPA ranking (multi-class classification) and GHS ranking (multi-class classification). EPA’s hazard classification [49] splits chemicals into four hazard categories: category I (LD_50_ ≤ 50 mg/kg) is the highest toxicity category; category II (moderately toxic) includes chemicals with 50 < LD_50_ ≤ 500 mg/kg; category III (slightly toxic) includes chemicals with 500 < LD_50_ ≤ 5000 mg/kg. Safe chemicals (LD_50_ > 5000 mg/kg) are included in Category IV.

To start with, the training set of 8890 compounds (Additional file 4) was released to the community for model development related to the EPA toxicity ranking endpoint. Then the test set of 48,138 compounds was released and predictions for these compounds were requested for each of the five endpoints. Finally, the evaluation set of 2896 compounds (Additional file 5), which were an undisclosed subset of the test set and whose values for all endpoints were known, was used to assess the submitted predictions and rate the corresponding models. All models submitted were rated and ranked by the NICEATM panel on the basis of several performance statistics [47].

The performance statistics for the TS and the ES are given below (Tables 3 and 4 respectively). For both datasets there was a positive correlation between performance measures (Accuracy, $\kappa$) and ADM (Fig. 4c, d). There was also very good concordance between the results for the TS and the ES. This is likely a consequence of the robust framework based on local models, chosen independently for each compound, which can mitigate some faults or errors of the dataset: such flaws will only affect a limited number of compounds rather than the whole ES.

**Table 3:**
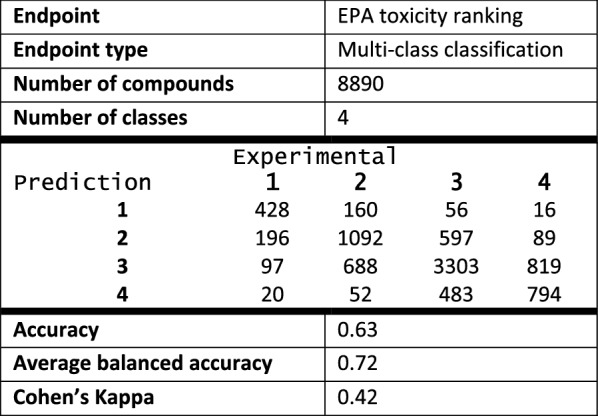
EPA acute oral toxicity ranking TS—statistics, confusion matrix and performance measures

Average balanced accuracy was calculated as an average of balanced accuracies for each class

**Table 4:**
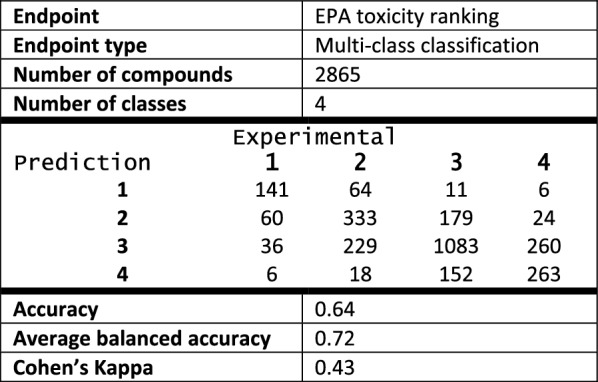
EPA acute oral toxicity ranking ES—statistics, confusion matrix and performance measures

Average balanced accuracy was calculated as an average of balanced accuracies for each class

## Conclusions

We have proposed a new methodology for predictive QSAR modelling based on local group selection during model development and at-the-runtime execution. The main feature of aiQSAR is compound-specific building of predictive models: for each target compound, only a local group of TS compounds that are structurally similar to the target is considered. We quantified this similarity in terms of the ADM, which is based on Tanimoto distances of molecular fingerprints. Three application examples are discussed: BCF for regression, Ames test for binary classification and EPA acute oral toxicity ranking for multi-class classification. The ADM positively correlated with performance metrics in all of them.

In general, we believe that this QSAR approach based on local group selection and modelling automation can raise the overall quality of in silico predictions. This could be especially important for certain endpoints with under-represented classes, where localization in a natural way, with no sampling required in balancing procedures, might improve the chemical space for QSAR predictions. We plan to fully develop this methodology into a stand-alone software tool that could easily be run on any dataset, without prior knowledge of the intricate details involved in QSAR modelling. This should help open up the field to a wider range of users.

## Additional files

**Additional file 1.** R scripts used to generate the models, along with input datasets, calculated descriptors and instructions to run the code and reproduce the results.

**Additional file 2.** Bioconcentration factor dataset and predictions.

**Additional file 3.** Ames test dataset and predictions.

**Additional file 4.** EPA toxicity ranking training set and predictions.

**Additional file 5.** EPA toxicity ranking evaluation set and predictions.

## Abbreviations

ADM: applicability domain measure
BCF: bioconcentration factor
EPA: Environmental Protection Agency
ES: evaluation set
FDA: Food and Drug Administration
$\kappa$: Cohen’s kappa coefficient
MAE: mean absolute error
NICEATM: NTP Interagency Center for the Evaluation of Alternative Toxicological Methods
QSAR: quantitative structure–activity relationship
RMSE: root-mean-square error
TS: training set

## References

[1] Gini G, Benfenati E (2016). QSAR methods. In silico methods for predicting drug toxicity.

[2] Cherkasov A (2014). QSAR modeling: where have you been? Where are you going to?. J Med Chem.

[3] Benfenati E (2010). The CAESAR project for in silico models for the REACH legislation. Chem Cent J.

[4] Gadaleta D (2018). QSAR modeling of ToxCast assays relevant to the molecular initiating events of AOPs leading to hepatic steatosis. J Chem Inf Model.

[5] Schultz TW, Cronin MTD, Walker JD, Aptula AO (2003). Quantitative structure—activity relationships (QSARs) in toxicology: a historical perspective. J Mol Struct.

[6] Tannenbaum J, Bennett BT (2015). Russell and Burch’s 3Rs then and now: the need for clarity in definition and purpose. J Am Assoc Lab Anim Sci.

[7] Gadatleta D (2017). Integrating computational methods to predict mutagenicity of aromatic azo compounds. J Environ Sci Health C.

[8] Roy K, Ambure P, Kar S, Ojha PK (2018). Is it possible to improve the quality of predictions from an “intelligent” use of multiple QSAR/QSPR/QSTR models?. J Chemom.

[9] Hewitt M (2007). Consensus QSAR models: Do the benefits outweigh the complexity?. J Chem Inf Model.

[10] Zhao C (2008). A new hybrid system of QSAR models for predicting bioconcentration factors (BCF). Chemosphere.

[11] Kleinstreuer NC (2018). Predictive models for acute oral systemic toxicity: a workshop to bridge the gap from research to regulation. Comput Toxicol.

[12] Fonseca NA, Rung J, Brazma A, Marioni JC (2012). Tools for mapping high-throughput sequencing data. Bioinformatics.

[13] Dong J (2017). ChemSAR: an online pipelining platform for molecular SAR modeling. J Cheminform.

[14] Soufan O (2018). DPubChem: a web tool for QSAR modeling and high-throughput virtual screening. Sci Rep.

[15] Chawla NV, Bowyer KW, Hall LO, Kegelmeyer WP (2002). SMOTE: synthetic minority over-sampling technique. J Artif Intell Res.

[16] O’Boyle NM (2011). Open Babel: an open chemical toolbox. J Cheminform.

[17] Gadaleta D, Lombardo A, Toma C, Benfenati E (2018). A new semi-automated workflow for chemical data retrieval and quality checking for modeling applications. J Cheminform.

[18] Ballabio D, Grisoni F, Todeschini R (2018). Multivariate comparison of classification performance measures. Chemom Intell Lab Syst.

[19] Ruili H, Menghang X (2017). Editorial: Tox21 challenge to build predictive models of nuclear receptor and stress response pathways as mediated by exposure to environmental toxicants and drugs. Front Environ Sci.

[20] Mayr A, Klambauer G, Unterthiner T, Hochreiter S (2016). DeepTox: toxicity prediction using deep learning. Front Environ Sci.

[21] Gunning D (2016) Explainable artificial intelligence (XAI). In: Program information. U.S. Defense Advanced Research Projects Agency. https://www.darpa.mil/program/explainable-artificial-intelligence. Accessed 02 Jan 2019

[22] Yuan H, Wang Y, Cheng Y (2007). Local and global quantitative structure—activity relationship modeling and prediction for the baseline toxicity. J Chem Inf Model.

[23] Martin T (2016) User’s guide for T.E.S.T. (version 4.2) (toxicity estimation software tool). U.S. Environmental Protection Agency

[24] Maunz A (2013). Lazar: a modular predictive toxicology framework. Front Pharmacol.

[25] Guha R, Dutta D, Jurs PC, Chen T (2006). Local lazy regression: making use of the neighborhood to improve QSAR predictions. J Chem Inf Model.

[26] Kode (2017) DRAGON 7.0.8

[27] Guha R (2007). Chemical informatics functionality in R. J Stat Softw.

[28] https://cran.r-project.org/web/packages/rcdk/rcdk.pdf. Accessed 02 Jan 2019

[29] ftp.ncbi.nlm.nih.gov/pubchem/specifications/pubchem_fingerprints.txt. Accessed 02 Jan 2019

[30] Faulon JL (2003). The signature molecular descriptor. 1. Using extended valence sequences in QSAR and QSPR studies. J Chem Inf Comput Sci.

[31] Bajusz D, Rácz A, Héberger K (2015). Why is Tanimoto index an appropriate choice for fingerprint-based similarity calculations?. J Cheminform.

[32] https://cran.rstudio.com/web/packages/fingerprint/fingerprint.pdf. Accessed 02 Jan 2019

[33] Kuhn M (2008). Building predictive models in R using the caret package. J Stat Softw.

[34] http://topepo.github.io/caret/available-models.html. Accessed 02 Jan 2019

[35] Hofner B, Mayr A, Robinzonov N, Schmid M (2014). Model-based boosting in R: a hands-on tutorial using the R package mboost. Comput Stat..

[36] Karatzoglou A, Smola A, Hornik K, Zeileis A (2004). Kernlab—An S4 package for kernel methods in R. J Stat Softw.

[37] Friedman J, Hastie T, Tibshirani R (2010). Regularization paths for generalized linear models via coordinate descent. J Stat Softw.

[38] Liaw A, Wiener M (2002). Classification and regression by randomForest. R News.

[39] Simm J, de Abril I, Sugiyama M (2014). Tree-based ensemble multi-task learning method for classification and regression. IEICE Trans Inf Syst.

[40] https://cran.r-project.org/web/packages/caTools/caTools.pdf. Accessed 02 Jan 2019

[41] https://cran.r-project.org/web/packages/e1071/e1071.pdf. Accessed 02 Jan 2019

[42] Hornik K, Buchta C, Zeileis A (2009). Open-source machine learning: R meets Weka. Comput Stat.

[43] https://cran.r-project.org/web/packages/sdwd/sdwd.pdf. Accessed 02 Jan 2019

[44] Benfenati E, Manganaro A, Gini G (2013). VEGA-QSAR: AI inside a platform for predictive toxicology. CEUR Workshop Proc.

[45] Landis WG, Sofield RM, Yu MH (2010). Introduction to environmental toxicology: molecular substructures to ecological landscapes.

[46] Mortelmans K, Zeiger E (2000). The Ames Salmonella/microsome mutagenicity assay. Mutat Res-Fund Mol M.

[47] ntp.niehs.nih.gov/pubhealth/evalatm/test-method-evaluations/acute-systemic-tox/models/index.html. Accessed 02 Jan 2019

[48] Walum E (1998). Acute oral toxicity. Environ Health Perspect.

[49] U.S. National Archives and Records Administration (2005) Toxicity category. In: Code of Federal Regulations. Office of the Federal Register. www.govinfo.gov/content/pkg/CFR-2005-title40-vol23/pdf/CFR-2005-title40-vol23-sec156-64.pdf Accessed 02 Jan 2019

